# Body Composition and Psychological Correlates of Resting Energy Expenditure and Activity in Adolescent Girls with Anorexia Nervosa

**DOI:** 10.3390/jcm15145738

**Published:** 2026-07-22

**Authors:** Stefano Lazzer, Lara Mari, Mattia D’Alleva, Jacopo Stafuzza, Simone Zaccaron, Nicola Campigotto, Enrico Rejc, Gabriella Tringali, Ilaria Grimoldi, Roberta De Micheli, Adele Bondesan, Anna Guerrini-Usubini, Alessandro Sartorio

**Affiliations:** 1 Department of Medicine, University of Udine, 33100 Udine, Italy; mari.lara@spes.uniud.it (L.M.); mattia.dalleva@uniud.it (M.D.); jacopo.stafuzza@uniud.it (J.S.); simone.zaccaron@uniud.it (S.Z.); nicola.campigotto@uniud.it (N.C.); enrico.rejc@uniud.it (E.R.); 2 School of Sport Sciences, University of Udine, 33100 Udine, Italy; 3 Department of Theoretical and Applied Sciences, eCampus University, Novedrate, 22060 Como, Italy; 4 Department of Neurosciences, Biomedicine and Movement Sciences, University of Verona, 37129 Verona, Italy; 5 Experimental Laboratory for Auxo-Endocrinological Research, Istituto Auxologico Italiano, Istituto di Ricovero e Cura a Carattere Scientifico (IRCCS), 28824 Piancavallo-Verbania, Italy; g.tringali@auxologico.it (G.T.); i.grimoldi@auxologico.it (I.G.); r.demicheli@auxologico.it (R.D.M.); a.bondesan@auxologico.it (A.B.); u.guerrini@auxologico.it (A.G.-U.); sartorio@auxologico.it (A.S.)

**Keywords:** anorexia nervosa, adaptive thermogenesis, indirect calorimetry, body composition, psychometric phenotypes

## Abstract

**Background**: Anorexia Nervosa (AN) triggers profound metabolic and psychological adaptations during adolescence. This study investigated the relationships between resting energy expenditure (REE), body composition, physical activity (PA), and specific psychometric phenotypes in adolescent girls with AN compared to healthy, normal-weight (NW) peers. **Methods**: Sixteen adolescent girls with severe AN (16.1 ± 1.2 year) and 16 age-matched NW controls (15.6 ± 1.5 year) underwent clinical evaluations: anthropometry, bioelectrical impedance analysis for fat-free mass (FFM) and fat mass (FM), indirect calorimetry for REE, the short-form International Physical Activity Questionnaire (IPAQ), and the Eating Disorder Inventory-3 (EDI-3). **Results**: Girls with AN exhibited significantly lower BMI (−20.3%, ES: 1.94, *large*), FFM (−18.3%, ES: 1.92, *large*), and FM (−25.7%, ES: 0.79, *medium*) than controls (*p* < 0.003). Absolute REE was drastically lower (−40.4%, ES: 3.53, *large*, *p* < 0.001). This hypometabolic state persisted after adjusting REE for body weight (−34.9%) or FFM (−32.5%, *p* < 0.001), consistent with adaptive thermogenesis. The AN group displayed resting hypotension and 79.0% (ES: 1.84, *large*, *p* < 0.001) lower total PA energy expenditure due to a lack of structured moderate-to-vigorous exercise; however, walking expenditure did not differ. Psychometrically, the AN cohort showed significantly higher (*p* < 0.001) values for drive for thinness (+316.9%, ES: 3.16, *large*), body dissatisfaction (+232.3%, ES: 2.30, *large*), and severe general psychological maladjustment, including higher interoceptive deficits (+148.2%, ES: 1.26, *large*). **Conclusions**: Adolescents with AN exhibit profound metabolic suppression via adaptive thermogenesis, together with an apparent dissociation between walking-related activity and hypotension. Since cognitive distortions and distress vary independently of anthropometric severity, clinical recovery must extend beyond nutritional rehabilitation; targeted psychotherapy may therefore be necessary to address persistent psychological maladjustment.

## 1. Introduction

Anorexia Nervosa (AN) is a severe psychiatric disorder characterized by persistent energy restriction, an intense fear of weight gain, and a distorted perception of body image [1]. Beyond its core psychological hallmarks, AN is defined by a state of profound, chronic energy deficiency that triggers systemic physiological adaptations [2]. Chief among these is the alteration in energy metabolism, which represents an evolutionary survival mechanism that preserves life during starvation [3]. Despite decades of clinical research, the precise interplay between resting energy expenditure (REE), body composition, and physical activity (PA) in adolescent AN patients remains a subject of ongoing debate [4].

Resting energy expenditure (REE) accounts for approximately 60% to 75% of total energy expenditure in sedentary individuals and is suppressed in patients with AN [5]. While this decline was historically attributed to the passive loss of metabolically active Fat-Free Mass (FFM) [6], current evidence indicates that the reduction in REE often exceeds that predicted by the loss of body weight alone [7]. This phenomenon, known as adaptive thermogenesis, represents a highly regulated increase in metabolic efficiency driven by neuroendocrine alterations, including suppressed triiodothyronine levels, diminished insulin-like growth factor 1, and decreased leptin [8].

This relationship is uniquely nuanced during adolescence, a critical physiological window characterized by rapid musculoskeletal development and hormonal stabilization [8]. In adolescent girls with AN, this developmental trajectory is severely disrupted; fat mass (FM) depletion and concomitant FFM catabolism serve to provide essential substrates for gluconeogenesis [9]. However, the preservation or restoration of FFM during nutritional rehabilitation is often inconsistent, and the specific contributions of distinct body compartments to the “metabolic sink” of adolescent patients require further clarification to optimize refeeding protocols [10]. Compounding this metabolic complexity is compulsive exercise or hyperactivity, a paradoxical feature observed in a significant subset of AN patients [11]. Despite severe energy deprivation, many adolescent girls exhibit high levels of PA, further exacerbating their energy deficit [12]. The current literature presents conflicting data regarding the metabolic impact of PA: some studies suggest that higher activity levels may “protect” against the full extent of metabolic slowdown by maintaining skeletal muscle mass [13], whereas others argue that the added energetic strain further depresses resting metabolism as a compensatory mechanism to minimize overall energy cost [14].

Furthermore, clinical management frequently encounters metabolic resistance during weight recovery, in which patients require unexpectedly high caloric intakes to sustain weight gain [15]. This highlights a dynamic, shifting relationship between REE and PA as the body transitions from acute starvation to nutritional restoration [16]. Because most of the existing data derive from adult cohorts or lack concurrent measurements of these variables, a significant knowledge gap persists regarding energy homeostasis in adolescents [17]. Precise metabolic profiling is critical to mitigate the risks of refeeding syndrome while ensuring caloric prescriptions are sufficient to support both weight restoration and linear growth [18].

These physiological and metabolic responses cannot be decoupled from the underlying psychological dimensions and behavioural phenotypes of the disorder [19]. Multidimensional tools like the Eating Disorder Inventory (EDI) provide essential quantifiable data on core eating disorder psychopathology—such as drive for thinness and body dissatisfaction—which directly influence the severity of dietary restriction and compulsive exercise [20]. Recent evidence [21], emphasizes that eating disorder psychopathology cannot be decoupled from broader psychological dimensions, including metacognitive dysfunction, moral emotions such as guilt, rigid self-monitoring, and profound interpersonal distrust. These psychological constructs often manifest clinically as heightened interpersonal insecurity, personal alienation, and asceticism. This suggests that psychological recovery requires an individualized therapeutic approach that extends far beyond simple weight and nutritional restoration.

The present study aimed to investigate the relationships between REE, body composition, and PA levels in adolescent girls diagnosed with AN compared to healthy peers. We hypothesized that girls with AN would show lower measured REE than controls even after adjustment for FFM, FM, age, and physical activity.

## 2. Materials and Methods

### 2.1. Participants

The study subjects were recruited from the Division of Eating and Nutrition Disorders at the Istituto Auxologico Italiano IRCCS (Piancavallo, Italy), a specialized tertiary care facility focused on the integrated treatment of severe obesity and eating disorders. Eligible participants were girls aged 13–18 years with a DSM-5 diagnosis of anorexia nervosa (AN) [1]. All participants in the AN group were classified as having the restrictive subtype. Mean illness duration was 3.4 ± 0.6 years. At the time of assessment, 12 participants had secondary amenorrhea, whereas 4 reported irregular menstrual cycles. Overall, 14 of 16 patients were receiving pharmacological treatment. The most frequently prescribed medication was sertraline (n = 10), followed by delorazepam (n = 7), aripiprazole (n = 6), olanzapine (n = 3), risperidone (n = 2), and melatonin (n = 2). No other regular medications were reported. Participants were assessed during the first week of admission, before the onset of significant nutritional rehabilitation. Conversely, individuals presenting with acute physical or psychiatric comorbidities that might interfere with study protocols were excluded from the study.

A control group of age-matched, normal-weight girls (NW) was identified by the research team from the School of Sports Science at the University of Udine. Given their regular engagement in recreational training sessions, this cohort is explicitly described and referred to as an active normal-weight (NW) comparison group.

### 2.2. Procedures and Data Collection

Following an informative briefing, written informed consent was obtained from the parents or legal guardians of all minor participants, alongside written assent from the adolescents themselves. An independent psychologist conducted an initial screening to verify compliance with the inclusion and exclusion criteria. It is noteworthy that all subjects invited to participate met the requirements and adhered to the protocol. To ensure objectivity, the outcome assessors were members of the research group who operated independently from the clinical care team. Data acquisition spanned from July 2024 to April 2026. The study protocol received formal approval from the Territorial Ethical Committee 5, Lombardy Region, Milan, Italy, on 23 April 2024 (reference code: 212/24; project code: 01C409). All research activities were conducted in strict adherence to the Declaration of Helsinki and its subsequent amendments regarding human experimentation.

#### 2.2.1. Anthropometric Assessments

Body weight (BW) was recorded using a standard scale (SECA, Hamburg, Germany), ensuring a precision of 0.1 kg. Standing height was measured using a Harpenden Stadiometer (Holtain Limited, Crymych, Dyfed, UK). Body mass index (BMI) was determined as body weight (kg) divided by height squared (m^2^).

#### 2.2.2. Body Composition Analysis

Body composition was evaluated using bioelectrical impedance analysis (Human-IM Scan, DS-Medigroup, Milan, Italy) according to the method of Lukaski et al. (1986) [22], after 20 min of rest in a supine position with relaxed arms and legs. To standardize hydration status, assessments were performed in the morning after voiding and an overnight fast, ensuring patients showed no clinical signs of peripheral edema or acute electrolyte imbalances. FFM and FM were determined in all subjects using the manufacturer’s validated prediction equations for adolescents.

#### 2.2.3. Resting Energy Expenditure

Resting Energy Expenditure (REE) was assessed following an overnight fast using an open-circuit, indirect computerized calorimetry system (Vmax 29, Sensor Medics, Yorba Linda, CA, USA) equipped with a rigid, transparent, and ventilated canopy. The calorimeter was calibrated prior to each test using standard gas mixtures (16% O_2_, 4% CO_2_). Ambient room temperature was strictly maintained between 22 °C and 24 °C. The total test duration was 30 min, preceded by a 5 min quiet rest period in a supine position. Steady state was defined as a minimum of 10 consecutive minutes during which variability in V’O_2_ and V’CO_2_ was <5%. Respiratory exchange ratios were checked continuously, and values outside the physiological range (0.70–1.00) served as exclusion criteria; however, no measurements were excluded. In participants with active, regular/irregular menses (n = 16 NW, n = 4 AN), testing was scheduled during the early follicular phase of the menstrual cycle; patients presenting with established secondary amenorrhea (n = 12 AN) were assessed upon admission regardless of timing. Energy expenditure was calculated from O_2_ uptake and CO_2_ output using the equation of Weir (1949) [23].

#### 2.2.4. Blood Pressure Assessment

Blood pressure readings were taken from the right arm using a sphygmomanometer (Gima, Gessate (MI), Italy) with an appropriately sized cuff while the subject was seated and relaxed. Measurements were taken three times at 5 min intervals, and the average of the three readings was recorded for both systolic (SBP) and diastolic (DBP) blood pressure.

#### 2.2.5. Blood Sample Collection

Blood samples were obtained from participants following a standardized procedure. Samples were drawn into lithium heparin tubes at approximately 8:00 AM after an overnight fast. Plasma was separated from blood cells via centrifugation (20–24 °C, 10 min at 2500 g) within two hours of collection. Then, the following biomarkers were measured: high-density lipoprotein cholesterol (HDL-C), triglycerides (TG) and glucose. Serum HDL-C and TG levels were assessed using colorimetric enzymatic assays (Roche Diagnostics, Monza, Italy), with sensitivities of 3.09 mg/dL [1 mg/dL = 0.03 mmol/L], and 8.85 mg/dL [1 mg/dL = 0.01 mmol/L], respectively. Serum glucose concentration was determined using the glucose oxidase enzymatic method (Roche Diagnostics, Monza, Italy), with a sensitivity of 2 mg/dL [1 mg/dL = 0.06 mmol/L]. The intra- and inter-assay coefficients of variation (CVs) were as follows: HDL-C: 1.8% and 2.2%; TG: 1.1% and 2.0%; Glucose: 1.0% and 1.3% (CRPRX, Roche Diagnostics GmbH, Mannheim, Germany) with a sensitivity of 0.03 mg/dL.

#### 2.2.6. Physical Activity Assessment

Physical activity was assessed using the short-form International Physical Activity Questionnaire (IPAQ), which focuses on the participant’s activity over the previous seven days [24]. The IPAQ is a standard instrument for monitoring physical activity across different populations and covers three main intensity domains: walking, moderate-intensity exercise, and vigorous-intensity exercise. Additionally, the questionnaire evaluates sedentary habits by tracking total sitting time. Participants record both the number of days and the daily duration spent on these activities during the prior week. This tool is widely recognized for its consistent measurement properties and its utility in comparing activity levels across various international settings.

#### 2.2.7. Eating Disorders

To evaluate broader eating disorder symptoms, the Eating Disorder Inventory-3 (EDI-3) was administered as a self-reporting instrument. This 91-item questionnaire explores specific dimensions of eating behaviour and psychological attitudes [20]. The subscales encompass diverse factors, including drive for thinness, bulimia, body dissatisfaction, and perfectionism, as well as more general psychological traits like ineffectiveness, interpersonal distrust, and social insecurity. Respondents rate each item on a six-point Likert scale that ranges from “always” to “never”. By summing the scores within each subscale, researchers can assess the severity of specific symptoms, with higher scores indicating a more significant presence of the trait in question.

### 2.3. Statistical Analysis

All statistical analyses were conducted using R software (version 4.5.2, The R Foundation for Statistical Computing, Vienna, Austria) and GraphPad Prism version 10.0.1 (IBM, Chicago, IL, USA).

Based on the reported difference in FFM between patients with anorexia nervosa (AN) and normal-weight controls (36.5 ± 3.3 vs. 40.5 ± 2.3 kg, respectively) [25], a sample size of 12 subjects per group is required to achieve a statistical power of 90% with a significance level α of 0.05, utilizing a two-tailed independent two-sample *t*-test assuming unequal variances. However, to account for potential measurement errors, the final sample size was increased to 16 subjects per group. Sample size calculations were performed using PASS 21 Power Analysis and Sample Size Software (2021) (NCSS, LLC, Kaysville, UT, USA).

Anthropometric, psychological, and physical activity variables were analyzed within a multivariate framework to compare individuals with anorexia nervosa (AN) and healthy controls (NW). The magnitude of between-group differences was interpreted using conventional Cohen’s d thresholds: small effect (d ≥ 0.20), medium effect (d ≥ 0.50), and large effect (d ≥ 0.80) [26,27].

Assumptions of linearity, homoscedasticity, and normality of residuals were verified using Shapiro–Wilk tests. When assumptions were violated, Welch’s *t*-test or non-parametric sensitivity analyses were considered. REE was analyzed using separate ANCOVA models including body weight or fat-free mass as covariates.

To test whether REE differences remain after adjustment, multiple linear regression models were constructed considering body composition, physical activity and psychological parameters:

Measured REE = group + FFM + FM + age + IPAQ + EDI composite

To reduce data dimensionality and obtain synthetic indices representative of each domain, Principal Component Analysis (PCA) was performed separately on anthropometric, physical activity and psychological variables after z-score standardization. The anthropometric component included BMI, FFM, and FM, resulting in an Anthropometric Score. Physical activity variables derived from the IPAQ questionnaire (walking, moderate, and vigorous activity) were combined into an IPAQ Score. The psychological component comprised the EDI-related subscales, generating a Psychological Score. The adequacy of the dataset for PCA was evaluated using the Kaiser–Meyer–Olkin measure and Bartlett’s test of sphericity. Components were retained based on eigenvalues > 1, scree plot inspection, and the proportion of explained variance. For each retained component, eigenvalues, percentage and cumulative explained variance, component loadings, and communalities were reported.

## 3. Results

No significant differences were observed between the two groups in age and stature (Table 1), confirming that the cohorts were well matched on demographic and developmental baselines.

As expected, subjects with AN exhibited significantly lower body weight and BMI (−20.3% and −20.9%; ES: 1.46, large and ES: 1.94, large; *p* < 0.001, respectively) compared to the NW group. Absolute FFM and FM were 18.3% (ES: 1.92, large) and 25.7% (ES: 0.79, medium) lower in the AN group than in the NW group (*p* < 0.003). Interestingly, when expressed as relative percentages of total body weight, neither FFM% (*p* = 0.377) nor FM% (*p* = 0.280) differed significantly between the groups (Table 1).

Absolute REE was significantly lower in the AN group compared to NW (−40.4%, ES: 3.53, large, *p* < 0.001). Crucially, this metabolic suppression persisted even after adjusting REE for body weight (−34.9%, *p* < 0.001) or FFM (−32.5%, *p* < 0.001) in adolescent girls with AN, suggesting a marked downregulation of metabolic efficiency per unit of FFM (Table 1).

A multiple linear regression model including group, FFM, FM, age, IPAQ score, and EDI composite score explained 83% of the variance in measured REE and was statistically significant (R^2^ = 0.83, F (11, 20) = 12.96, *p* < 0.001). Group was a significant independent predictor of measured REE (β = −627.9, 95% CI: −952.5 to −303.4, *p* < 0.001), and FFM was also positively associated with measured REE (β = 25.72, 95% CI: 0.28 to 51.2, *p* = 0.047). FM, age, IPAQ score, and EDI composite score were not significant predictors.

**Figure 1:**
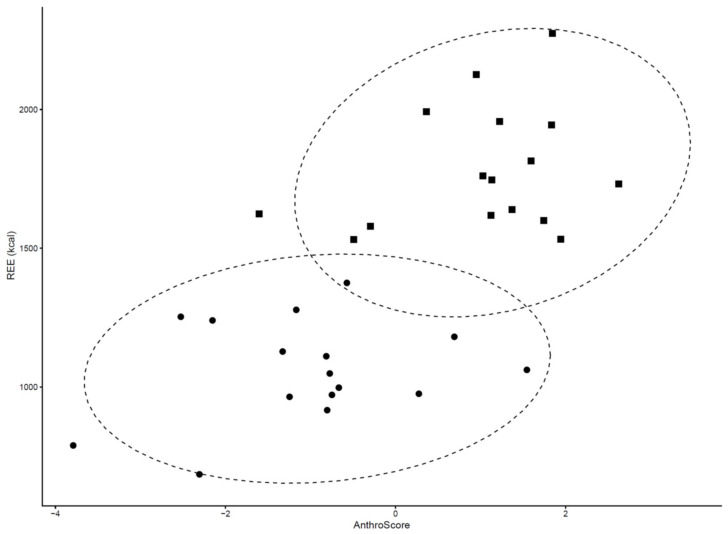
Association between Resting Energy Expenditure (REE) and composite anthropometric score (AnthroScore: body mass index, fat free mass, fat mass) across girls with anorexia nervosa (AN, ●) and normal weight controls (NW, ■). r = 0.70, *p* < 0.001, with 95% confidence ellipses shown.

Figure 1 shows that body composition (BMI, FFM, FM) was a primary correlate with REE. The AN group exhibited lower REE, partly in association with reduced body-size compartments. The Kaiser–Meyer–Olkin measure indicated adequate sampling adequacy for PCA (KMO = 0.58), and Bartlett’s test of sphericity was significant (χ^2^ = 62.5, df = 3, *p* < 0.001), supporting the factorability of the correlation matrix. Principal component analysis identified one component with an eigenvalue greater than 1.0. This component explained 81.1% of the total variance. The second and third components explained 15.4% and 3.5% of the variance, but their eigenvalues were below 1.0.

Both systolic and diastolic blood pressures were significantly lower in the AN group than in the control group (by −13.6 and −19.0%; ES: 1.75, large, and ES: 2.59, large, respectively, *p* < 0.001, Table 2), underscoring a state of generalized resting hypotension. Conversely, no statistically significant differences were observed between the two groups in HDL-cholesterol, triglycerides, and plasma glucose levels (Table 2).

Regarding physical activity, total energy expenditure expressed in metabolic equivalents (IPAQ_TOT) was significantly lower in the AN group than in the NW group (−79.0%, ES: 1.84, large, *p* < 0.001, Table 3). This discrepancy was primarily driven by the structured exercise reported by the NW group. Specifically, the AN group showed a reduction of 87.0% in vigorous physical activity (IPAQ_VIG, ES: 0.24, small, *p* < 0.001) and 86.6% in moderate physical activity (IPAQ_MOD, ES: 1.18, large, *p* = 0.002) compared to NW, who reported performing three weekly sessions of recreational training. Conversely, low-intensity activity, measured as walking metabolic expenditure (IPAQ_WALK), showed no statistically significant difference between the two groups and did not suggest major group-level response bias.

**Figure 2:**
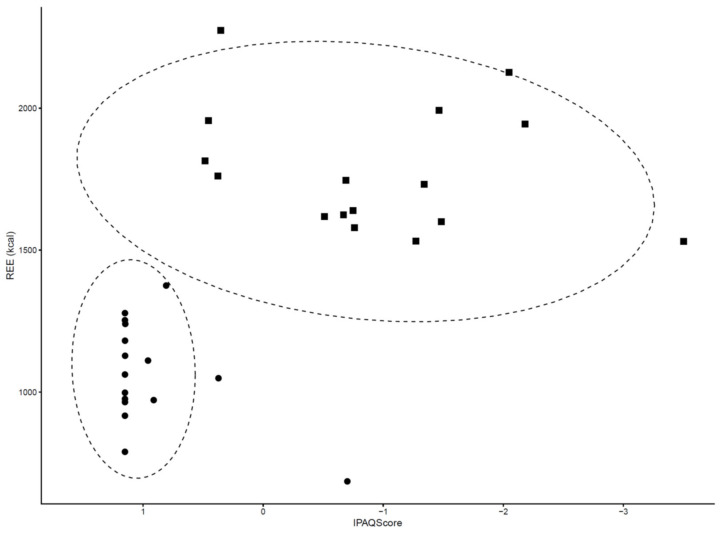
Association between Resting Energy Expenditure (REE) and composite IPAQ score (walking, moderate and vigorous activity) across girls with anorexia nervosa (AN, ●) and normal weight controls (NW, ■) r = 0.57, *p* < 0.001, with 95% confidence ellipses shown.

Figure 2 shows that the AN group has a severely suppressed REE and generally scores lower on the physical activity index (IPAQ) compared to the NW group, resulting in two distinct physiological profiles. The KMO measure showed acceptable sampling adequacy for PCA (KMO = 0.49), and Bartlett’s test of sphericity was significant (χ^2^ = 13.3, df = 3, *p* < 0.004), supporting the factorability of the correlation matrix. Principal component analysis identified three components with an eigenvalue greater than 1.0. The first component explained 53.3%, while the second 33.6% and the third 13.1% of the total variance.

The most pronounced and clinically relevant discrepancies emerged within the eating disorder risk scales (Table 3). The AN group exhibited a 316.9% (ES: 3.16, large) higher drive for thinness and a 232.3% (ES: 2.30, large) higher score in body dissatisfaction, compared to the NW group (*p* < 0.001). These marked increases confirm that dysmorphic ideation and a restrictive drive constitute the prominent psychopathological correlates within the AN group. Conversely, scores for the Bulimia subscale did not differ significantly between the two groups, suggesting that in the AN group binge-eating and purging behaviours are minimal or absent.

AN group presented (Table 3) severe deficits in identity and self-esteem, as evidenced by a 232.1% (ES: 2.16, large) higher low self-esteem and a 200.0% (ES: 1.49, large) higher score (*p* < 0.001) in personal alienation. Relational dynamics were similarly impaired, with the AN group reporting a 98.8% (ES: 1.16, large) higher interpersonal insecurity and a 103.0% (ES: 1.06, large) higher interpersonal alienation (*p* < 0.005). These metrics clinically translate into social withdrawal and attachment difficulties. Furthermore, a 148.2% higher (ES: 1.26, large, *p* < 0.001) in interoceptive deficits highlighted a compromised capacity to interpret internal visceral and emotional cues. This was paired with a 162.5% (ES: 1.43, large, *p* < 0.001) higher asceticism and a 30.7% (ES: 0.74, medium, *p* < 0.05) higher maturity fears, delineating a classic phenotype of rigid self-denial and developmental anxieties. Interestingly, emotional dysregulation and perfectionism failed to reach statistical significance.

These patterns were reflected in the composite scores, which corroborated the overall severity of the condition (Table 3). The eating disorder risk composite and the inadequacy composite were 231.7% (ES: 2.64, large) and 221.0% (ES: 1.99, large) higher in the AN group, respectively (*p* < 0.001). Most notably, the global general psychological maladjustment composite score was 103.2% (ES: 1.56, large, *p* < 0.001) higher in the AN group, indicating a substantial overall psychological burden in this population.

**Figure 3:**
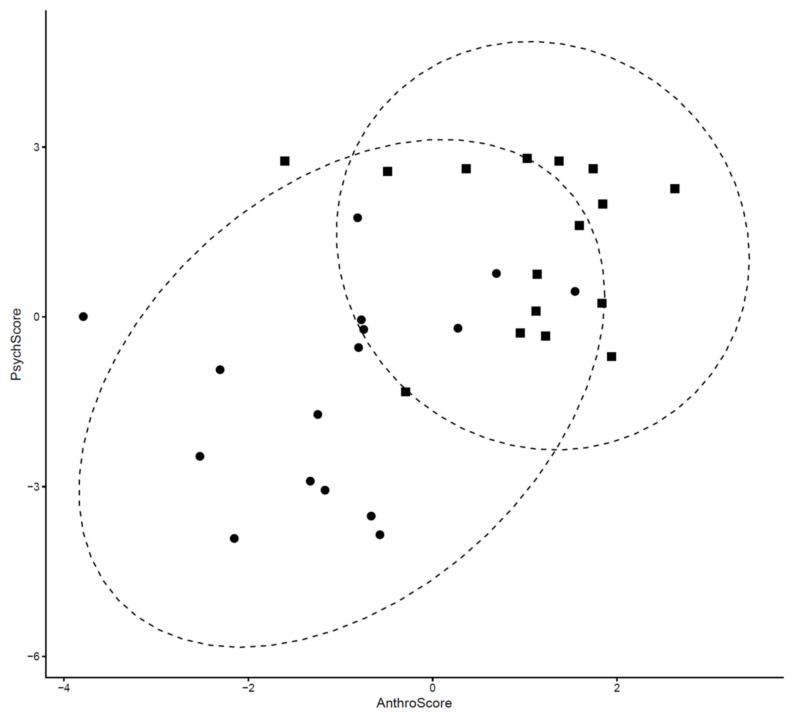
Association between eating disorder score (PsychScore: EDI-3) and composite anthropometric score (AnthroScore: body mass index, fat free mass, fat mass) across girls with anorexia nervosa (AN, ●) and normal weight controls (NW ■). r = 0.50, *p* < 0.003, with 95% confidence ellipses shown.

The bivariate analysis (Figure 3) reveals a clear distinction between the AN and NW groups, driven by concurrent physical and psychological variations. While the NW group maintains high, stable psychological scores across normal anthropometric (BMI, FFM and FM) ranges, the AN group displays symptoms and psychological traits alongside tissue depletion. The KMO statistic indicated acceptable sampling adequacy for PCA (KMO = 0.57). Bartlett’s test of sphericity was statistically significant (χ^2^ = 243.0, df = 10, *p* < 0.001), indicating that the correlation matrix was suitable for principal component analysis. Principal component analysis retained one component with eigenvalues greater than 1.0. This component accounted for 85.5% of the total variance.

Importantly, from a methodological standpoint, the response style validity indicators (Inconsistency, Infrequency, and Negative Impression, Table 3) did not differ significantly between groups and did not suggest major group-level response bias.

## 4. Discussion

The results of this study provide a comprehensive, multidimensional characterization of the physiological, metabolic, and psychological landscapes that define adolescent girls with AN compared with NW peers. By utilizing a tightly matched demographic baseline (with no significant differences in age and stature), the observed deviations reflect the broader clinical expression of chronic severe energy restriction and psychological distress.

As expected, the phenotypic hallmark of the AN cohort is a profound reduction in body weight (−20.3%) and BMI (−20.9%). This depletion spans both absolute FFM (−18.3%) and FM (−25.7%). Interestingly, when converted to relative percentages (FFM% and FM%), the differences failed to reach statistical significance. This indicates a proportional, systemic shedding of both adipose and metabolic tissues, a protective biological approach observed during prolonged starvation states to preserve vital organ functions [28]. The most striking physiological finding relates to metabolic adaptation. The absolute REE in the AN group was severely depressed (−40.4%). Crucially, this metabolic suppression cannot be explained solely by the loss of metabolically active tissue; when adjusting REE for body weight (−34.9%) or FFM (−32.5%), the hypometabolic state persisted with high significance (*p* < 0.001). In healthy populations, FFM serves as the primary mathematical correlation with REE, as visually corroborated by the control group in Figure 1. However, in the AN group, there is a true downregulation of metabolic efficiency per unit of FFM. This biological phenomenon, is highly suggestive of a hypometabolic profile compatible with metabolic adaptation, which represents an extreme energy-conservation mechanism [29]. To prevent death from starvation, the body downregulates energy-expensive cellular processes, primarily through suppression of the thyroid hormone axis, leptin, and sympathetic nervous system activity [3,30]. Because circulating endocrine markers (such as T3 or leptin) were not directly measured in this protocol, these findings should be interpreted as compatible with adaptive thermogenesis rather than confirming specific underlying neuroendocrine pathways.

The cardiovascular data closely mirror the hypometabolic profile. While fundamental biochemical homeostasis was maintained—demonstrated by the lack of significant differences in serum HDL-cholesterol, triglycerides, and plasma glucose—the hemodynamic profile reveals a distinct pattern. The AN cohort exhibited generalized resting hypotension, with systolic and diastolic pressures depressed by −13.6% and −19.0%, respectively. This presentation represents a bradycardic and hypotensive adaptation to minimize cardiac workload and conserve thermal and chemical energy [2].

Regarding physical activity, the following paradox emerged: total energy expenditure from physical activity (IPAQ_TOT) was drastically lower in the AN group (−79.0%), which was heavily driven by a near-total absence of structured vigorous (−87.0%) and moderate (−86.6%) exercise. This sharp decline aligns with the three weekly sessions of recreational training reported by the NW group. Conversely, low-intensity ambulatory activity (IPAQ_WALK) showed no significant difference between the groups. This “walking paradox” is consistent with the clinical literature on compulsively exercise and hyperactivity in AN [31], showing that patients may maintain high levels of low-intensity non-exercise activity thermogenesis, such as restless walking or pacing, even when severely malnourished and medically hypotensive [32,33]. This behaviour is driven by an obsessive urge to expend energy combined with neurobiological alterations in reward pathways [32]. This distinct behavioural-metabolic profile is clearly summarized by the complete separation of clusters in Figure 2. From a methodological standpoint, however, the short-form IPAQ is structurally limited because it measures general physical activity volume without capturing these compulsive motivations or subtle forms of light non-exercise activity thermogenesis [34]. This limitation reflects a broader trend in the literature, where self-report tools in AN populations frequently misclassify or underestimate light activity when compared to objective accelerometery.

The psychometric data paint an equally severe picture, confirming that the physical state is tethered to profound psychological implications. Results showed higher values in drive for thinness (+316.9%) and body dissatisfaction (+232.3%) in AN cohort, while the bulimia subscale remained statistically unaltered. This specific psychometric profile aligns with classic EDI validation data, which identify elevated drive for thinness and body dissatisfaction alongside baseline bulimia scores as the hallmark characterization of purely restrictive phenotypes [20,35]. Beyond core eating disorder risks, the EDI-3 general scales reveal systemic psychological maladjustment (+103.2%). The clinical phenotype is characterized by a collapsing sense of self, quantified by severe deficits in self-esteem (+232.1%), high personal alienation (+200.0%) and marked interoceptive deficits (+148.2%). Together, these findings suggest broader metacognitive and interpersonal dysfunctions, characterized by rigid self-evaluation, excessive self-monitoring, and interpersonal distrust, which may contribute to maintaining restrictive behaviours. Likewise, elevated asceticism may reflect a tendency toward moral self-denial, whereby restrictive behaviours become associated with self-worth or relief from guilt. The elevated interoceptive deficits score is particularly relevant to the physiological data; it highlights a severely compromised capacity to interpret internal visceral, metabolic, and emotional cues accurately. This biological-cognitive disconnect explains how patients can ignore severe hunger, hypothermia, and exhaustion [36,37]. Furthermore, elevations in asceticism (+162.5%) and maturity fears (+30.7%) outline a rigid framework of self-denial and developmental anxiety. Interestingly, emotional dysregulation and perfectionism did not reach statistical significance. This may suggest that, in this specific adolescent cohort, the acute distress associated with dysmorphic ideation and starvation could outweigh the influence of broader personality traits. This phenomenon aligns with current cognitive-interpersonal models, which suggest that the acute neurobiological consequences of severe starvation and body image distress can temporarily overshadow or homogenize broader, long-standing personality constructs in younger patients [38]. The bivariate analysis in Figure 3 synthesizes the physical and psychological findings. While the healthy control group maintains a tight, stable, and resilient psychological profile across normal anthropometric ranges, the AN cohort displays a wide, vertically elongated distribution of profound psychological symptoms alongside tissue depletion. This visual elongation highlights that while tissue depletion is relatively uniform under starvation, the corresponding psychological fragmentation is highly heterogeneous and uncoupled from absolute somatic severity. This phenomenon underscores the complex network-like interaction between physical wasting and psychological vulnerability in clinical phenotypes [39]. The extensive vertical elongation of the AN cluster indicates that psychological impairment varies widely and independently of the immediate physical severity (Figure 3). This highlights a critical clinical consideration: weight restoration alone does not guarantee psychological recovery. Nutritional rehabilitation may normalize anthropometric characteristics and restore suppressed REE; however, persistent psychological features—such as alienation, low self-esteem, and interoceptive deficits—require targeted, long-term therapeutic interventions to achieve full clinical remission [20].

Several limitations of the current study must be acknowledged. First, the sample size is relatively small (n = 32 total) and may limit statistical stability, especially for psychological subscales and PCA-derived indices. Therefore, these findings should be interpreted as exploratory and require confirmation in larger cohorts. Second, body composition was assessed via BIA rather than DXA, which may introduce measurement variability related to hydration status especially in severely underweight individuals with AN. Third, physical activity was recorded via a self-reported questionnaire (IPAQ short-form) rather than objective accelerometery, creating a vulnerability to under-reporting or misclassifying light activity, and no specific compulsive exercise scales were utilized. Fourth, the control group consisted of active peers, which may overestimate physical activity differences relative to the general adolescent population. Finally, critical metabolic biomarkers (e.g., T3, leptin, IGF-1) were unavailable for adjustment.

## 5. Conclusions

This study highlights the multi-systemic features of adolescent anorexia nervosa, demonstrating that profound REE suppression and psychological maladjustment coexist. Behaviourally, the findings suggest a potential preservation of low-intensity ambulation despite resting hypotension and marked energetic depletion. Crucially, because cognitive distortions and interoceptive deficits vary independently of anthropometric severity, physical weight restoration alone cannot be assumed to equate to complete clinical recovery. Longitudinal studies are required to determine how resting energy expenditure, objective activity patterns, and specific psychometric symptoms change and interact during long-term nutritional and therapeutic recovery.

## Figures and Tables

**Table 1:** Anthropometric characteristics, body composition and resting energy expenditure of girls with anorexia nervosa (AN) and normal weight (NW).

	AN (n = 16)	NW (n = 16)	*p*-Value
Age (year)	16.1 ± 1.2	15.6 ± 1.5	0.257
Stature (m)	1.64 ± 0.07	1.63 ± 0.08	0.670
Body weight (kg)	43.7 ± 7.6	54.8 ± 7.6	0.001
Body mass Index (kg/m^2^)	16.3 ± 2.5	20.6 ± 1.9	0.001
Fat-free mass (kg)	33.6 ± 4.1	41.1 ± 3.7	0.001
Fat Mass (kg)	10.1 ± 4.5	13.6 ± 4.4	0.003
Fat-free mass (%)	76.9 ± 7.6	75.7 ± 5.7	0.377
Fat Mass (%)	23.1 ± 7.8	24.3 ± 5.7	0.280
REE (kcal/die)	1061 ± 182	1779 ± 222	0.001
REE adjusted BW (kcal/die)	1119 (54)	1721 (54)	0.001
REE adjusted FFM (kcal/die)	1145 (55)	1696 (55)	0.001

Data are presented as mean ± standard deviation or (standard error). *p* values were obtained using independent-samples *t*-tests. REE was analyzed using separate ANCOVA models including body weight (BW) or fat-free mass (FFM) as covariates. REE: Resting Energy Expenditure.

**Table 2:** Blood pressure and metabolic outcomes of girls with anorexia nervosa (AN) and normal weight (NW).

	AN (n = 16)	NW (n = 16)	*p*-Value
Systolic blood pressure (mmHg)	98.6 ± 12.0	114.1 ± 3.6	0.001
Diastolic blood pressure (mmHg)	63.1 ± 7.9	77.9 ± 1.7	0.001
HDL-cholesterol (mg/dL)	62.2 ± 11.7	57.2 ± 4.7	0.122
Triglycerides (mg/dL)	72.5 ± 21.6	75.3 ± 5.8	0.626
Glycemia (mg/dL)	72.1 ± 9.0	75.6 ± 5.0	0.176

Data are presented as mean ± standard deviation. *p* values were obtained using independent-samples *t*-tests.

**Table 3:** Results from International Physical Activity Questionnaire (IPAQ) and Eating Disorder Inventory-3 (EDI-3) questionnaire of girls with anorexia nervosa (AN) and normal weight (NW).

	AN (n = 16)	NW (n = 16)	*p*-Value
***International Physical Activity Questionnaire***			
IPAQ_TOT (MET-min/week)	738.3 ± 1130.8	3516.0 ± 1810.8	0.001
IPAQ_VIG (MET-min/week)	225.0 ± 730.4	1737.0 ± 834.9	0.001
IPAQ_MOD (MET-min/week)	181.3 ± 303.4	1350.0 ± 1369.3	0.002
IPAQ_WALK (MET-min/week)	332.1 ± 772.6	429.0 ± 515.1	0.679
***Eating Disorder Inventory-3***			
*Eating disorder risk scales:*			
Drive for thinness	24.6 ± 5.4	5.9 ± 6.4	0.001
Bulimia	4.1 ± 3.9	2.8 ± 3.2	0.284
Body dissatisfaction	31.9 ± 10.2	9.6 ± 9.1	0.001
*General psychological scale:*			
Low self-esteem	17.6 ± 5.8	5.3 ± 5.6	0.001
Personal alienation	14.4 ± 7.7	4.8 ± 4.9	0.001
Interpersonal insecurity	17.1 ± 7.1	8.6 ± 7.6	0.003
Interpersonal alienation	13.4 ± 6.4	6.6 ± 6.4	0.005
Interoceptive deficits	20.6 ± 11.2	8.3 ± 8.0	0.001
Emotional dysregulation	9.7 ± 5.4	6.6 ± 4.9	0.103
Perfectionism	9.8 ± 5.3	6.6 ± 5.2	0.090
Asceticism	12.6 ± 6.5	4.8 ± 4.2	0.001
Maturity Fears	19.6 ± 6.6	15.0 ± 5.8	0.046
*Composite scores:*			
Eating disorder risk composite	60.7 ± 15.6	18.3 ± 16.4	0.001
Inadequacy composite	32.1 ± 12.2	10.0 ± 9.9	0.001
Interpersonal problems composite	30.6 ± 12.8	15.2 ± 13.5	0.002
Affective problems composit	30.3 ± 15.4	14.9 ± 11.3	0.003
Overcontrol composite	22.4 ± 10.6	11.3 ± 7.6	0.002
General psychological maladjustment composite	134.9 ± 47.5	66.4 ± 40.3	0.001
*Response style validity indicators:*			
Inconsistency	6.8 ± 4.0	6.3 ± 4.1	0.762
Infrequency	21.8 ± 13.5	39.5 ± 54.6	0.230
Negative impression	8.6 ± 3.8	16.6 ± 18.5	0.103

Data are presented as mean ± standard deviation. *p* values were obtained using independent-samples *t*-tests.

## Data Availability

Raw data will be uploaded to www.zenodo.org immediately after the acceptance of the manuscript and they will be available upon reasonable request to the authors S.L. and A.S.

## Abbreviations

The following abbreviations are used in this manuscript:

AN	Anorexia Nervosa
BD	Body Dissatisfaction
BMI	Body Mass Index
BW	Body Weight
DBP	Diastolic Blood Pressure
DT	Drive for Thinness
EDI-3	Eating Disorder Inventory-3
FFM	Fat-Free Mass
FM	Fat Mass
HDL-C	High-Density Lipoprotein Cholesterol
IPAQ	International Physical Activity Questionnaire
NW	Normal-Weight
PA	Physical Activity
PCA	Principal Component Analysis
REE	Resting Energy Expenditure
SBP	Systolic Blood Pressure
TG	Triglycerides
